# 298. A Novel Murine Model of Neonatal *Staphylococcus aureus C*olonization Induces Changes in the CD4 T cell Compartment and Protects from Lethal Bacteremia

**DOI:** 10.1093/ofid/ofad500.370

**Published:** 2023-11-27

**Authors:** Patrick D Olson, Juliane Bubeck-Wardenburg

**Affiliations:** Washington University School of Medicine, Saint Louis, Missouri; Washington University School of Medicine in St. Louis

## Abstract

**Background:**

*Staphylococcus aureus* is a leading infectious cause of invasive bacterial infection in the United States. The pathogen’s capacity to cause disease is due, in part, to its ability to asymptomatically colonize the human host. Classically, the nose has been presumed to be the primary colonization site, but there is increasing evidence that the gastrointestinal tract may be a fundamental niche occupied during *S. aureus* colonization. There remain significant deficits in preclinical modeling of invasive *S. aureus* infection; Laboratory mice are commonly employed for modeling of *S. aureus* infection, but are rarely colonized by or pre-exposed to *S. aureus*. This contrasts with the human interaction with the pathogen, where a vast majority of humans demonstrate a specific adaptive response to *S. aureus* and a plurality of patients have lifelong colonization. To overcome this deficit, we designed a novel neonatal mouse model of asymptomatic intestinal *S. aureus* colonization.

**Methods:**

Neonatal mice were subjected to oral gavage with *S. aureus* at day 10 of life; colonization was followed by fecal *S. aureus* titer. Infection outcomes were compared between mock- and *S. aureus* colonized mice.

**Results:**

Mice from internal breeding and external vendors had no detectable *S. aureus* in the nasopharynx or feces at all time points tested. We found that adult mice are unable to be durably colonized by *S. aureus* despite multiple methods of inoculation. We identified that neonatal mice have a narrow, early window of susceptibility when they can be durably colonized, demonstrating robust fecal titers of *S. aureus* sustained through adulthood. Intestinal exposure to *S. aureus* shifted the nature of the colonic T cell compartment to encompass a greater proportion of CD4 T regulatory cells and a decreased frequency of GATA3^+^ CD4 cells. Finally, colonized mice demonstrated protection from severe infection, mimicking what is seen in humans.

**Conclusion:**

Our findings support the hypothesis that gastrointestinal colonization influences the development of systemic immunity against *S. aureus.* This model will allow exploration into the mechanism of protective immunity against *S. aureus* and may inform the development of novel therapeutic and vaccination approaches to mitigate staphylococcal disease.

**Disclosures:**

**All Authors**: No reported disclosures

